# Treatment Process and Participant Characteristic Predictors of Substance Use Outcome in Mentorship for Addiction Problems (MAP)

**DOI:** 10.4172/2329-6488.1000171

**Published:** 2014-08-28

**Authors:** Kathlene Tracy, Deborah Guzman, Mark Burton

**Affiliations:** NYU Department of Psychiatry, NYU School of Medicine, USA

**Keywords:** Peer support, Alcohol, Drug, Outcome predictors, Behavioral treatment

## Abstract

There are a variety of self-help treatments which have components of sponsorship or peer support. Although there has been a recent surge in the utilization of peer support interventions within clinical settings, there is limited data on substance use outcome predictors for interventions designed solely for peer support within community treatment settings that are empirically based. We examined both treatment process and participant characteristic variables as predictors of substance use outcomes within our Stage I pilot which developed a new intervention, Mentorship for Addiction Problems (MAP). We found treatment process variables to be significantly associated with substance use outcome and no participant characteristic variables.

## Introduction

Although data exists within the literature on variables associated with substance use outcome within various forms of self-help treatments that have components of sponsorship or peer support [1,2], there is limited data on substance use outcome predictors when delivering empirically based treatment designed solely for peer support within community treatment settings. With the rise in adoption of utilizing peer support services in clinical settings that often have not been empirically tested, studies are needed to begin to understand what key components of the peer support treatments are associated with better outcome and whether or not the treatments afford greater benefits to certain subpopulations [3–6].

One of the more documented self-help treatment process variables associated with decreased substance use and abstinence is attendance. Individuals who attended Alcoholics Anonymous (AA) more often are more likely to maintain abstinence from alcohol than those who attended less frequently [7–10]. Similar findings occurred in Narcotics Anonymous (NA), where lower levels of alcohol use and marijuana use are reported with more consistent attendance [11,12]. However, engagement may also play a role, as those who frequently engage in 12-Step activities, but attend meetings inconsistently are shown to have better drug use outcomes than individuals who did not regularly engage in 12-Step activities, but attended consistently [13].

Additionally, individual characteristics may affect substance use outcomes in self-help interventions that include peer support. Individuals with substance use disorders and Posttraumatic Stress Disorder (PTSD) who attended AA more often are more likely to remain abstinent than those with only substance use disorders [14]. However, the type of co-occurring diagnoses may make a difference on outcome impact. Individuals who had depression and a substance use disorder have a weaker association between self-help involvement and abstinence than individuals with substance use disorders alone [15]. This was the impetus for the emergence of dually focused 12-Step programs, such as Double Trouble, which specifically address the needs of individuals with co-occurring disorders [16,17].

While treatment process and individual characteristics seem to play a role in substance use outcome when involved in self-help programs that include a peer supportive element, the peer sponsorship activities were not regulated in amount delivered across participants or rigorously tested. To begin to address this, we examined both treatment process and participant characteristic predictors of substance use outcomes within our Stage I pilot that developed a new intervention, Mentorship for Addiction Problems (MAP), for individuals with substance use disorders by pilot/feasibility testing, manual writing, training program development, adherence and competence measure construction. Similar to other Stage I pilot investigations, this pursuit will also help to begin to understand potential active ingredients within MAP and potential participant characteristics that may contribute to outcome to inform directions in future larger trials to further substantiate results and develop the treatment. This work was supported by the National Institute on Alcohol Abuse and Alcoholism (NIAAA) (R01AA016160) and the National Institute on Drug Abuse (NIDA) (R34DA034898).

## Materials and Methods

Within the Stage I study, 40 clients (10 mentors and 30 mentees) were selected for participation from a chemical dependency outpatient clinic at Bellevue Hospital Center to develop a new intervention, Mentorship for Addiction Problems (MAP), for individuals with substance use disorders by pilot/feasibility testing, manual writing, training program development, adherence competence measure construction in an uncontrolled trial. There were two cohorts of 20 participants. For each cohort, a pool of 5 mentors engaged in mentoring activities for 24 weeks (6 months) until 15 recently admitted mentees participated in MAP for 12 weeks (3 months). The study was approved by the governing institution’s human subjects review board prior to the initiation of the pilot.

### Measures

The Structured Clinical Interview for DSM-IV Axis I Disorders (SCID-I) was administered to participants to assess lifetime and/ or current psychiatric diagnoses. Mentors met lifetime diagnosis for a substance use disorder and were at least 6 months abstinent from drugs and alcohol. Mentees met current diagnosis of a substance use disorder, were actively using substances, and were recruited during the first 3 months of treatment when vulnerable to relapse and attrition are high.

Behavioral and biological measures were conducted at baseline, weekly, monthly, and termination for all participants during the study. The behavioral assessments included: 1) Diagnostic - SCID-I/ P and Clinician Abstinence Verification (CAV). 2) Demographic - Demographic Form (Demo). 3) Alcohol/Drug **-**Addiction Severity Index – Lite (ASI-Lite), Substance Clinical Global Impression Scale (SCGI-O)-Observer, (SCGI-S)–Self, Substance Use Report (SUR), and Goal Attainment Scaling Rating (GAS). 3) Safety **-** Adverse Events Form (AE). 4) Role Interactions **-** Quality of Life Inventory (QOLI), Non-Substance Social Contact (NSS), Making Decisions Scale (MDS), General Self Efficacy Scale (GSE), Coping Behaviors Inventory (CBI), Multifactor Leadership Questionnaire (MLQ). 5) HIV-Risk Behavior **-** Risk Behavior Survey (RBS). 6) TAU Fidelity/Non Study Treatment Activity **-** Program and Client Cost-Substance Abuse Treatment (PACC-SAT), Non Study Peer Support Scale (NSPS), Medication Adherence Scale (MAS), and Supervisor Tape Rating Form (STRF). 7) MAP Attendance – mentorship group attendance sign-in sheets and supervision group attendance sign-in sheets. Screening assessments included: SCID and CAV. Baseline, monthly, and termination assessments included: Demo, ASI-Lite, SCGI-O, SCGI-S, MDS, MLQ, RBS, and STRF (with the exception of the demographics form which was only done at baseline). The weekly assessments included the NSS, SUR, PACC-SAT, GSE, CBI, NSPS, and MAS. The biological measures included a breathalyzer test and a 5 panel urine toxicology measure; testing the substances MDMA, opiates, methamphetamine, cocaine, and marijuana.

In addition to the above assessments, adherence and competence measures were also utilized throughout the study to ensure that the intervention was being delivered correctly and completely. The Mentorship Adherence and Competence Scale (MACS) was based on the Yale Adherence and Competence Scale (YACS) [18] and revised for MAP. It was utilized to rate the adherence and competence of the Mentor’s delivery of MAP during the tape recorded introductory mentorship pair meeting. The Mentorship Contact Log (MCL) was used to record the mentors/mentees contact outside of the group. It captures the date of contact, type, location, amount of time spent and contents. It was completed weekly throughout the treatment. The third form of fidelity measure, the Mentorship Rating Scale (MRS), was developed from modifying a validated mentorship scale utilized in therapeutic communities for assessment within MAP [19]. It asks the rater to indicate how much they agree with statements related to the mentor’s delivery of MAP treatment in the following areas: MAP activities, ability to rely on mentor, role modeling and awareness of alcohol/substance use and actions. It was completed by both the mentor and mentee weekly throughout treatment. The last form of fidelity measure, the Mentor Supervisory Review (MSR), asks the supervisor to indicate how much they agree with statements related to variables that are thought to contribute to a successful mentoring: communication and impact of actions, initiator, motivator for abstinence and emotional control. Initially it was completed beginning, mid, and end of the study to isolate the most useful time to deliver scale during the treatment.

### Focus groups

There were four focus groups completed with participants. Clinician feedback surveys were also completed with each participant’s clinician to obtain satisfaction and additional feasibility data on the MAP. There were six clinicians within the chemical dependency outpatient unit and the 40 research participants for this study were from their caseloads. Within the focus groups and clinician surveys information was obtained on MAP’s impact on participants (e.g., taking steps toward becoming or remaining abstinent, managing or reducing psychiatric symptoms, reaching work-related goals, treatment connection, improved social support networks) and satisfaction with MAP’s program structure (e.g., mentor/mentee pairing being overall ‘good fits’, pair selection process, level of supervision provided, mentorship activities, frequency and quantity of mentorship contact, level of interference with regular treatment).

### MAP theoretical foundation

The main focus of the mentoring within MAP is the development of a relationship based on abstinence by rapport building. The foundation for MAP is rooted within the Community Reinforcement Approach (CRA). CRA has demonstrated the importance of valued social roles in maintaining abstinence [20,21]. Having a relationship based on abstinence not only provides reinforcement for the mentee who often has limited relationships outside of those based on alcohol/substance use in early recovery, but also provides a valued social position for the mentor who is seen as a role model to help achieve and sustain abstinence.

We further built upon this relationship by utilizing Goal Attainment Scaling (GAS) to guide the interactions. The mentor collaborates with the mentee to help them achieve abstinence goals using harm reduction strategies which are monitored through modified Goal Attainment Scaling (GAS) [22–24]. GAS was originally developed to evaluate community programs [25,26], but then later was adopted as an outcome measure to assess progress of patient-specific goals and effectiveness of treatments [22–24]. We utilized the development and rating of GAS plans in MAP to measure the mentees progress on goals to remain abstinent throughout treatment and to provide guidelines for the communications. Having this structure to define the mentorship relationship contributed to its success while reducing occurrences of boundary problems.

### MAP treatment components

MAP has four key components: 1) Mentorship Training that lasts for 1 hour 2 times per week for 4 weeks where the mentors learn the characteristics of being an effective mentor and how to embody these characteristics while working within a structure of GAS recovery plans. Rather than condensing the training to one day of training, we chose to have potential mentors come twice a week for 4 weeks to begin to allow for a commitment to the process. If individuals are unable to keep these scheduled appointments, it is likely that a similar occurrence would happen when making commitments to the mentee. 2) Weekly Mentorship Group that is co-facilitated by the supervisory clinician with mentors and includes the mentees lasting 1 hour 1 time per week. Entailed in the group are discussions of development of GAS recovery plans, monthly formal mentee presentations of the progression these plans and weekly discussions to receive guidance and support from mentors and the other members of the group to achieve goals of these plans. Mentees can use this time to receive collective guidance from all of the mentors in addition to it providing another venue for the supervisory clinician to view the mentorship relationship and provide guidance. The groups are based on a progression of role development and change supported by comradeship. 3) Individual Pair Contact that includes 1 to 4 hours of mentor mentee pair contact per week which is either over the phone or in-person and includes general support regarding achieving goals in the GAS recovery plans. In-person individual contacts may include non-substance using social activities (e.g. going for coffee, home visitation, meeting at the clinic, going to the movies, attending family social functions) or treatment-related activities (e.g. attending Alcoholics Anonymous, taking to treatment appointments, assisting with housing searches). 4) Supervision occurs throughout the program to ensure ethical standards are being held. There is a weekly mentorship supervisory group held 1 hour prior to the weekly mentorship group; informal supervisory interaction during the ongoing weekly mentorship group; review of mentorship delivery adherence/competence rating forms; mid-point meetings with the supervisory clinician and mentor; and supervision on and hoc basis if any problems emerge needing immediate attention.

### Additional details and planned analyses

For additional details on any of the above procedures, please see the main outcome publication which resulted from the study by Tracy and colleagues [6]. A series of bivariate regressions were conducted on select variables based on the literature to begin to explore whether any treatment process or participant variables were associated with substance use outcomes.

## Results

Sixty-seven patients were approached to be in the program and 52 (78%) patients signed informed consent with 40 (77%) participants entering the study. Patients who did not proceed further in the process were excluded due to not meeting the entry criteria for the study. Of the 40 participants who entered the study, there were only 4 who dropped out yielding a 90% retention rate. All drop outs were mentees.

Fifteen (38%) of the participants were females. Sixteen participants (40%) were African American or Black, 15 (38%) White, and 9 (22%) Hispanic. Ages ranged from 19 to 70 years with a mean of 50.3, SD=9.97. Table 1 presents a comparison of demographic and diagnostic variables by mentees and mentors.

Forty participants (100%) had a diagnosis of past or current alcohol abuse or dependence. All mentors had lifetime diagnoses. Twenty-nine participants (73%) had an alcohol abuse or dependence diagnoses in addition to a co-occurring mood disorder diagnosis and 12 (30%) had a co-occurring anxiety disorder diagnosis. There was a broad range of other substance use disorders, 25 (62%) cocaine, 21 (53%) cannabis, 19 (48%) opioid, 10 (25%) poly, 1 (2%) sedative hypnotics, and 1 (2%) other substances.

All participants were in similar stages of their recovery and enrolled in an inner city community outpatient treatment program that matched recovery stage to required groups and individual treatment. In addition to weekly group therapy and individual meetings with their substance abuse counselor, participants who were prescribed psychiatric medication or had mental health needs met with an attending psychiatrist monthly for treatment.

The main outcome paper presents the promising data on consistency in treatment across participants, safety, significant reductions in alcohol and drug use, high participant and clinician satisfaction with the MAP, and strong adherence/competence to the delivery of the treatment. Thus, these data will not be presented in this paper as the scope is to present results on the treatment process and participant variables associated with substance use outcome.

A series of bivariate regressions were conducted for the following 6 treatment process variables: 1) mentor supervision group attendance, 2) mentee mentorship group attendance, 3) mentor mentorship group attendance 4) total mentorship contact hours for mentee mentor pair, 5) total phone contact hours for mentee mentor pair, and 6) total face-to-face contact hours mentee mentor pair. Table 2 presents a breakdown of these treatment process variables by percent day’s abstinent drugs and alcohol, drugs only, and alcohol only for mentees. Mentor supervision group attendance, mentee mentorship group attendance, and mentor mentorship group attendance were shown to be predictive of percentage of days abstinent from substances for mentees. Attendance was recorded on the group attendance sign-in sheets. Total mentorship contact hours, total phone contact hours, and total face-to-face contact hours were not associated percentage of days abstinent from substances for mentees. Contact hours were assessed through the MCL. Abstinence data was derived from self-report data from the SUR verified by the objective urine toxicology and breathalyzer results.

A second series of bivariate regressions were conducted for the following common participant characteristic variables: 1) gender, 2) ethnicity (i.e., African American or Black, American Indian or Alaskan, Asian or Pacific Islander, Caucasian or White, Latino), 3) age, 4) homelessness, 5) substance severity, 6) psychiatric treatment, 7) absence or presence of SCID-I major depression disorder, and 8) absence or presence of SCID-I Post-Traumatic Stress Disorder (PTSD). Homelessness was defined by not having housing/being declared homeless at any point during participation within the study. Substance severity was defined by the total number of years of primary drug use. Psychiatric treatment was defined by total number of times in outpatient and inpatient treatment. Table 3 presents a breakdown of these participant characteristic variables by percent day’s abstinent drugs and alcohol, drugs only, and alcohol only for mentees with none of the variables being significant.

## Discussion

With the rise in utilization of peer mentorship interventions to augment substance abuse treatment programs, it becomes increasingly more important to investigate the treatment process and participant characteristics that may impact substance use outcomes when implementing peer support interventions that are empirically based. In knowing potential predictors of substance use outcomes, peer support programs can be tailored to highlight key components of a treatment and selection if necessary of subpopulations that may achieve greater benefits from involvement. In addition, in preliminary analyses of new interventions, these results can be utilized to guide the further investigation and development of the treatment.

This was the first exploration of patient and treatment process variables associated with substance use outcome within Mentorship for Addictions Problems (MAP). MAP is a new peer supportive intervention that formalizes client to client mentorship relationships as an adjunct to standard substance abuse outpatient treatment. It is comprised of selection, training, and supervision procedures that enable successful recovering patients to serve as mentors for clients who are early in the recovery process. MAP is conceived as an optional module that can be incorporated into professionally run treatment programs based on a wide range of treatment philosophies.

We found the following MAP treatment process variables; mentor supervision group attendance, mentee mentorship group attendance, and mentor mentorship group attendance to be associated mentee percent days abstinent in our initial analyses. This underscores the likely importance of the components of treatment being delivered in MAP in substance use outcomes with better outcomes when the MAP supervision and weekly treatment groups are utilized. Mentors had a goal which they met of interacting with the mentee at least 1 to 4 hours per week in the study; however, there was a range across mentors of amount of mentorship provided to mentees. Therefore, we were interested in seeing if the amount of mentorship over and above the goal predicted substance use outcomes and there was not an association. Similarly, we examined whether in person or phone contact were associated with outcome and they were not. Thus, in our preliminary analyses, it does not appear to be useful to stipulate the mechanism for communication.

We found none of the participant characteristics that we investigated in our initial analyses to be associated with abstinence. Neither gender, ethnicity, age, homelessness status, substance severity, number of psychiatric treatments, PTSD diagnosis, or Major Depression diagnosis were associated with substance use outcome. This begins to support the overall usefulness in MAP treating a broad range of patients from varying backgrounds and functioning levels.

While these data are promising in beginning to explore key variables associated with substance use outcome in an empirically based peer support treatment, MAP, it should be noted that these results are derived from a Stage I study. Future larger randomized studies are needed to further substantiate the results with preliminary studies such as this pilot being utilized to inform the direction of these trials and the further development of MAP.

## Figures and Tables

**Table 1:** Comparison of Demographic Variables by Mentor and Mentee

	Mentees (N=30)	Mentors (N=10)
Gender Females	11 (37%)	4 (40%)
Males	19 (63%)	6 (60%)
Age	M=48.9, Range 19 to 70	M=54.5, Range 48 to 63
Ethnicity African American or Black	13 (43%)	3 (30%)
American Indian or Alaskan	0	0
Asian or Pacific Islander	0	0
Caucasian or White	11 (37%)	4 (40%)
Latino	6 (20%)	3 (30%)
Psychiatric Disorder Mood	20 (66%)	9 (90%)
Anxiety	10 (33%)	2 (20%)
Psychotic	0	0
Substance Use Disorder Alcohol	30 (100%)	10 (100%)
Cannabis	16 (53%)	5 (50%)
Cocaine	18 (60%)	7 (70%)
Opioid	13 (43%)	6 (60%)
Homeless	9 (30%)	2 (20%)

**Table 2:** Treatment Process Predictors

Mentee Percentage of Days Abstinence			
Treatment Process Variable	Drugs and Alcohol	Drug Only	Alcohol Only
Mentor Supervision Group Attendance	R^2^ =.790, F(1,8) =30.13, *** p<.001	R^2^=.458, F(1,8)=6.76, * p<.05	R^2^= .393, F (1,8)=393, p=.053
Mentee Mentorship Group Attendance	R^2^=.228, F(1,28)=8.28, * p<.05	R^2^=.056, F(1,28) =1.647, p=.210	R^2^=.158, F(1,28)=5.27, * p<.05
Mentor Mentorship Group Attendance	R^2^=.798, F(1,8)=31.50, *** p<.001	R^2^=.462, F(1,8)=6.76, * p<.05	R^2^=. 362, F(1,8) =1.71, p=.171
Phone Contract Hours	R^2^=.009, F(1,28)=.252, p=.620	R^2^=.017, F(1,28)=.481, p =.494	R^2^=. 001, F(1,28) =.039, p=.844
Face-To-Face Contact Hours	R^2^=.012, F(1,28)=.346, p=.561	R^2^=.013, F(1,28)=.375, p=.545	R^2^=. 001, F(1,28) =.039, p=.275
Total Contact Hours	R^2^=.011, F(1,28)=.373, p=.574	R^2^=.008, F(1,28)=.213, p=.648	R^2^=. 037, F (1,28) =1.07, p=.308

Significant Finding Indicated by following:

* p<.05;

** p<.01;

*** p<.001

**Table 3:** Participant Predictors

Mentee Percentage of Days Abstinence			
Participant Variable	Drugs and Alcohol	Drug Only	Alcohol Only
Gender	R^2^=.001, F(1,28)=31.50, p=.842	R^2^=.000, F(1,28)=.000, p=.967	R^2^=. 001, F(1,28) =.025, p=.875
Ethnicity	R^2^=.010, F(1,28)=.600, p=.600	R^2^=.076, F(1,28)=.222, p=.141	R^2^=. 003, F(1,28) =.095, p=.760
Age	R^2^=.021, F(1,28)=.015, p=.445	R^2^=.113, F(1,28)=3.55, p=.070	R^2^=. 001, F (1,28) =.015, p=.905
Homelessness	R^2^=.018, F(1,24)=.431, p=.518	R^2^=.028, F(1,24)=.699, p=.411	R^2^=. 008, F(1,24) =.198, p=.660
Substance Severity	R^2^=.001, F(1,28)=.028, p=.869	R^2^=.003, F(1,28)=.088, p=.769	R^2^=. 000, F(1,28) =.011, p=.917
Psychiatric Treatment	R^2^=.002, F(1,28)=.051, p=.822	R^2^=.033, F(1,28)=6.76, p=.285	R^2^=.001, F(1,28) =31.50, p=.848
Major Depression	R^2^=.051, F(1,28)=1.06, p=.314	R^2^=.000, F(1,28)=.001, p=.982	R^2^=. 078, F(1,28) =2.07, p=.207
PTSD	R^2^=.008, F(1,28)=.224, p=.640	R^2^=.016, F(1,28)=.452, p=.507	R^2^=. 041, F(1,28) =.046, p=.832

Significant Finding Indicated by following:

* p<.05;

** p<.01;

*** p<.001
